# *Aspergillus fumigatus* cerebral abscess and sphenoid sinus osteomyelitis in an immunocompetent patient following previous nasopharyngeal carcinoma and radiotherapy

**DOI:** 10.1093/jscr/rjab402

**Published:** 2021-09-22

**Authors:** Bernard J H Kim, Joseph Garcia Redmond, Annabelle D Donaldson, A Robert Aspoas

**Affiliations:** Department of Neurosurgery, Auckland City Hospital, Auckland, New Zealand; Department of Neurosurgery, Auckland City Hospital, Auckland, New Zealand; Department of Infectious Diseases, Auckland City Hospital, Auckland, New Zealand; Department of Neurosurgery, Auckland City Hospital, Auckland, New Zealand

## Abstract

Cerebral mycosis is extremely rare in immunocompetent patients. A 61-year-old male presented with a 3-month history of worsening left-sided headaches and 3-week history of left-sided upper lip paraesthesia. Magnetic resonance imaging revealed an enhancing lesion in the left temporal lobe. Histopathology of this lesion revealed what initially resembled a zygomycete but additional cultures obtained on further surgical debridement revealed the infection to be *Aspergillus fumigatus* with associated sphenoid sinus osteomyelitis. We postulate that the presentation was related to the patient’s previous radiotherapy for nasopharyngeal carcinoma. To the best of our knowledge, this is the only report of such a case.

## INTRODUCTION

Cerebral mycosis is extremely rare in immunocompetent patients. It normally afflicts those who are compromised due to immunosuppressants, solid organ and hematological malignancies, or infections such as human immunodeficiency virus [1]. Mortality rates are high despite advancements in neurosurgery and anti-fungal therapy [2]. Even in the immunocompetent, mortality rates can be as high as 66–90% [3].

## CASE REPORT

A 61-year-old Chinese male presented with a 3-month history of worsening left-sided headaches and 3-week history of left-sided upper lip paresthesia. He had these symptoms intermittently over the course of the year, but they had recently worsened. Due to his past medical history of migraines, previous presentations to hospital were dismissed as sumatriptan overuse. In 2001, he had been diagnosed with nasopharyngeal carcinoma (WHO Type III) in the left fossa of Rosenmüller. The nasopharynx and base of skull were treated with 70 Gy 35 fractions, while the neck received 60 Gy 30 fractions. Follow-up over 5 years showed no residual or recurrent tumor.

At presentation, neurological exam was normal aside from a known left-sided blindness since childhood and on-going paresthesia of V2 region of cranial nerve V. The patient was afebrile and all other observations were within normal limits.

**Figure 1:**
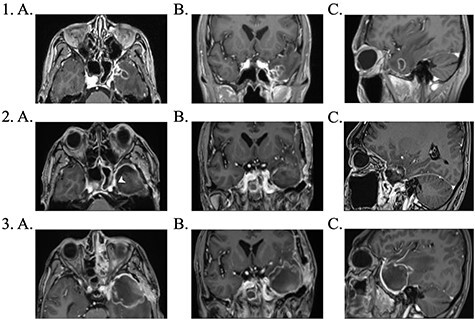
1. Pre-operative contrast enhanced brain MRI: (**a**) T1 axial view with enhancing lesion; (**b**) T1 coronal and (**c**) T1 sagittal. 2. First post-operative contrast enhanced brain MRI showing resection of lesion: (**a**) T1 axial. Arrow: dural enhancement of sphenoid sinus; (**b**) T1 coronal and (**c**) T1 sagittal. 3. Second post-operative contrast enhanced brain MRI following endoscopic re-resection: (**a**) T1 axial; (**b**) T1 coronal and (**c**) T1 sagittal.

Brain magnetic resonance imaging (MRI) study with contrast revealed an irregular enhancing mass within the left temporal lobe measuring 3 × 2.6 × 2cm with significant surrounding parenchymal oedema (Fig. 1). In addition, enhancement of the adjacent dura extending to the left temporal fossa, pterygoid muscle and left sphenoid sinus was observed.

**Figure 2:**
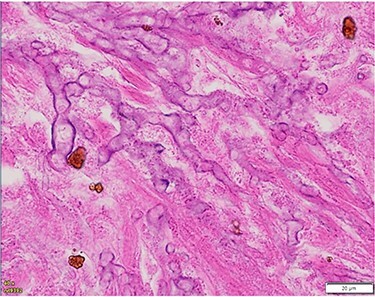
Histopathology of left temporal lobe lesion resembling zygomycete but later confirmed *A. fumigatus*.

The patient underwent a left-sided temporal lobectomy to excise the lesion but, due to the proximity to the cavernous sinus, the lesion was not followed beyond the dural attachment on the middle fossa floor. Unexpectedly, the histopathology revealed hyphae resembling a zygomycete, with broad (5–12 μm) hyphae without septae and branching angles between 45° and 90° (Fig. 2). Liposomal amphotericin B treatment was initiated at 5 mg/kg per day and later increased to 10 mg/kg per day. Fungal cultures yielded no growth.

The patient underwent further endoscopic re-resection a week following his initial operation. The sphenoid sinus appeared necrotic and a frank collection of fungal matter was observed in the space. Fungal culture from this repeat surgery grew *Aspergillus fumigatus* and histology was consistent with this diagnosis, demonstrating narrow, septated hyphae with branching angles around 45°. The lateral wall of the sphenoid sinus revealed signs of osteomyelitis from chronic *Aspergillus* infection. There was no evidence of mucormycosis from any culture. Following sensitivity reports, the antifungal agent was changed to voriconazole due to acute kidney injury from liposomal amphotericin B.

Further surgical debridement was undertaken due to concerns on MRI of intracerebral abscess formation. The appearance at theatre was that of a hematoma, and bacterial cultures yielded no growth.

Investigations for auto-immune conditions, hematological malignancies, diabetes mellitus and other immuno-compromising conditions were negative. At time of discharge, the patient was clinically stable.

Due to liver function derangement from voriconazole, the antifungal agent was changed to posaconazole. The patient completed a total of 12 months antifungal therapy. Subsequent serial MRI imaging showed good response to therapy with no further recurrence.

## DISCUSSION

Fungal cultures confirmed that our patient had an *Aspergillus* cerebral abscess with associated osteomyelitis. This has a poor prognosis with a crude mortality rate of 25%. The sphenoid sinus may have been more susceptible to infection due to destruction following treatment of previous nasopharyngeal carcinoma [4]. Given dural enhancement around the lateral aspect of the sinus and extension into the temporal lobe, it is most likely this bony compromise provided the tract for cerebral invasion.

Invasive aspergillosis of the immunocompetent patient is extremely rare. Best outcome is observed with extensive surgical resection followed by long-term antifungal therapy [5, 6]. Our report emphasizes the importance of fungal culture in confirming the diagnosis. Due to the inherent friability of hyphae, the yield from fungal culture may be very low and treatment for mucormycosis is often initiated based on histopathology [7]. However, tissue examination does not always reliably differentiate between hyphae of morphologically similar species, especially chronic *Aspergillus* infections [8]. The correct diagnosis is vital for both prognosis and guiding treatment. Following culture results, and the additional complication of renal impairment from liposomal amphotericin, the antifungal was changed to voriconazole as studies have shown it to be more effective and less toxic [9, 10]. This is thought to be due to its increased ability to penetrate the central nervous system [11] and it has been shown to improve 12-week survival rates from 57.9 to 70.8% [10]

Invasive fungal sinusitis can be broadly divided into acute, chronic and granulomatous types [12]. Patients with chronic infections often present with rhinosinusitis, nasal obstruction and/or purulent discharge. Our patient had none of these typical symptoms due to the sphenoid sinus being infected. For such sinonasal infections, fungal production of elastase resulting in thrombotic occlusion can cause territorial infarcts in vessels around the cavernous sinus [13].

In addition to these symptoms, imaging can help with early diagnosis. In the immunocompromised, lesions appear thick and irregular with heterogenous ring-like enhancement on MRI due to abscess cavity [14]. However, for immunocompetent individuals, they are more likely to be solid, homogenous and contrast enhancing lesions with paranasal sinus involvement. Dural enhancement is usually seen adjacent to the infected paranasal sinuses, which represents a direct extension of the infection, as was the case with our patient [15]. Computed tomographic imaging is largely non-specific with the fungal mass appearing hyperintense due to calcium salts and metal ions deposited in necrotic areas [1, 13].

We present the first case of an immunocompetent patient with cerebral invasion of *A. fumigatus* with associated sphenoid sinus osteomyelitis following previous radiotherapy. Although invasive fungal infections in the immunocompetent are rare, our case demonstrates the importance of considering it in the differential diagnosis of a cerebral lesion in the presence of relevant risk factors. With radiotherapy frequently used in the treatment of nasopharyngeal malignancies, this potential complication needs to be recognized.

## CONFLICT OF INTEREST STATEMENT

The authors report no conflict of interest or any funding.

## FUNDING

None.
